# Immunotoxicity and Transcriptome Analyses of Zebrafish (*Danio rerio*) Embryos Exposed to 6:2 FTSA

**DOI:** 10.3390/toxics11050459

**Published:** 2023-05-15

**Authors:** Jing Zhang, Zongming Ren, Meng Chen

**Affiliations:** Institute of Environment and Ecology, Shandong Normal University, Jinan 250358, China; 15506686558@163.com

**Keywords:** 6:2, fluorotelomer, sulfonic acid, zebrafish, embryo, immunotoxicity, transcriptome analyses

## Abstract

As a new alternative to perfluorooctane sulfonic acid (PFOS), 6:2 fluorotelomer sulfonic acid (6:2 FTSA) has been widely produced and used in recent years, and its concentration and frequency of detection in the aquatic environment and aquatic organisms are increasing. However, studies of its toxicity in aquatic biological systems are alarmingly scarce, and the relevant toxicological information needs to be improved. In this study, we investigated AB wild-type zebrafish (*Danio rerio*) embryos subjected to acute 6:2 FTSA exposure for immunotoxicity using immunoassays and transcriptomics. Immune indexes showed significant decreases in SOD and LZM activities, but no significant change in NO content. Other indexes (TNOS, iNOS, ACP, AKP activities, and MDA, IL-1β, TNF-α, NF-κB, TLR4 content) all showed significant increases. These results indicated that 6:2 FTSA induced oxidative stress and inflammatory responses in zebrafish embryos and exhibited immunotoxicity. Consistently, transcriptomics showed that genes involved in the MAPK, TLR and NOD-like receptor signaling pathways (*hsp70*, *hsp701*, *stat1b*, *irf3*, *cxcl8b*, *map3k8*, *il1b*, *tnfa* and *nfkb*) were significantly upregulated after 6:2 FTSA exposure, suggesting that 6:2 FTSA might induce immunotoxicity in zebrafish embryos through the TLR/NOD-MAPK pathway. The results of this study indicate that the safety of 6:2 FTSA should be examined further.

## 1. Introduction

Perfluorooctane sulfonate (PFOS), an organic fluoride with hydrophobic and oleophobic properties and high stability, is widely used in firefighting, food and chemical production and other industries [1,2,3,4]. Studies have found that PFOS is widespread in various environmental media around the world, and is hazardous to biological health and the ecological environment, so the production and use of PFOS and its derivatives have been restricted or banned in several countries and regions [1,5,6,7,8,9]. However, because the application of PFOS in industrial production and daily life is considered essential, PFOS alternatives have been developed [10,11,12,13]. The use of 6:2 fluorotelomer sulfonate (6:2 FTSA) as a substitute represents an improvement on the structure of PFOS, replacing part of the C-F bond with a C-H bond to improve water solubility while ensuring similar physical and chemical properties to PFOS, and is currently being used as a PFOS replacement on a large scale in the European market [14,15]. This is expected to lead to new environmental pollution problems. Indeed, recent studies have detected 6:2 FTSA in the water environment and aquatic organisms, and that the detection rate and concentration of this substance are on the rise [16,17]. For example, the average concentration of 6:2 FTSA detected in seawater samples from the edge area of China was only 0.14–0.16 ng/L, but the detection rate reached 100% [18,19]. In the point source pollution water of an urban area in Melbourne, Australia, 6:2 FTSA became the locally dominant pollutant, with the average concentration reaching as high as 3000 ± 670 ng/L [17]. Furthermore, 6:2 FTSA has been detected in fish and even human placenta in many other places, including Canada and Sweden, and the concentration of 6:2 FTSA detected in European sea bass samples (<0.025–2.2 ng/g ww) was even higher than that of PFOS (<0.025–0.93 ng/g ww) [20,21,22].

Although it is generally accepted that 6:2 FTSA exhibits a low enrichment capacity in aquatic organisms, and it has been suggested that its bioconcentration factor in zebrafish embryos (*Danio rerio*) is much lower than that of PFOS [23]. Exposure to 6:2 FTSA has been found to induce behavioral toxicity in zebrafish, resulting in a significant increase in swimming distance under dark conditions [24]. There have been a large number of studies on the toxicity of PFOS in biological development, immunity, endocrines, reproduction and other forms of toxicity [25,26,27]. Considering the structural similarity between 6:2 FTSA and PFOS, the question of whether 6:2 FTSA produces similar toxicity to PFOS is worth exploring. However, toxicological studies on 6:2 FTSA in aquatic organisms are scarce, and additional studies are urgently needed. The immune system is an important barrier for the body to achieve self-protection against external stimuli. Under exposure to environmental pollutants, organisms may experience a series of immune responses such as oxidative stress and inflammation. PFOS has been shown to induce oxidative stress and lipid peroxidation damage in zebrafish, amplifying the inflammatory response by interfering with the nuclear factor-κB (NF-κB) signaling pathway, thereby triggering immunotoxicity in zebrafish [28]. Additionally, it has also been shown that 6:2 FTSA exposure leads to a significant increase in inflammatory response-related proteins in adult male mice, causing liver inflammation [16]. Based on the above studies, we hypothesized that 6:2 FTSA may also induce immunotoxicity in aquatic organisms.

In this study, zebrafish embryos were used as a model to explore the immunotoxicity of 6:2 FTSA in aquatic organisms. The immunotoxicity of acute exposure to 6:2 FTSA in zebrafish embryos was evaluated by combining the change trends of immune-related biochemical indexes and immune-related proteins. Additionally, transcriptome analysis combined with Gene Ontology (GO) and Kyoto Encyclopedia of Genes and Genomes (KEGG) enrichment analysis was used to explore the internal mechanism of immunotoxicity induced by 6:2 FTSA in zebrafish embryos. The results of this study will provide basic information to guide future studies of 6:2 FTSA immunotoxicity in aquatic organisms.

## 2. Materials and Methods

### 2.1. Preparation of Chemicals and Test Solutions

6:2 FTSA (CAS No. 27619–97–2, purity ≥ 98%) was purchased from Beijing Bailingwei Technology Co., Ltd. (Beijing, China). The 6:2 FTSA stock solution (concentration, 0.2 g/L) was prepared with dimethyl sulfoxide (DMSO) as a co-solvent, and then diluted with fresh culture medium (400 mL of distilled water contains 0.0888 g of calcium chloride (CaCl_2_), 0.0241 g of magnesium sulfate (MgSO_4_), 0.0252 g of sodium bicarbonate (NaHCO_3_) and 0.0022 g of potassium chloride (KCl)) to a 0.5 mg/L exposure solution (0.25% DMSO). The other chemicals and reagents used in this study were of a high-performance liquid chromatography grade.

### 2.2. Adult Zebrafish Rearing and Embryo Collection

Adult AB wild-type zebrafish were purchased from the National Zebrafish Resource Center (Wuhan, China) and were raised in a standardized circular culture system of the Institute of Environment and Ecology, Shandong Normal University (Jinan, China) at a temperature of 28 ± 2 °C, light intensity of 4000 Lux, light and dark period of 14:10 h, in aerated water (pH: 7.8 ± 0.2; hardness: 250 ± 25 mg/L; conductivity: 525 ± 25 µS/cm), and were spot-fed twice a day. Adult male and female zebrafish were bred separately and fed three times a day one week before the experiment. The night before breeding, male and female zebrafish (female: male = 1:1) were placed in hatching boxes separated by partitions. In the morning of the next day, the partition was removed and the zebrafish were stimulated by light to encourage them to finish spawning. The process lasted for 2 h. After spawning, the adult zebrafish were put back into the tank for recovery, and the zebrafish embryos were collected in Petri dishes. Zebrafish embryos were rinsed with culture medium and incubated in a constant temperature-light incubator (Longyue, Shanghai, China) (28 ± 1 °C). Healthy individuals at 2 days post fertilization (dpf) were selected under an optical anatomic microscope for subsequent exposure experiment (exposure time: 72 h, exposure concentration: 0.5 mg/L). All of the animal procedures were approved by the ethical committee clearance, and the accession number is AEECSDNU2021079.

### 2.3. Experimental Design

6:2 FTSA concentrations were designed at 0.5 mg/L based on the results of our preliminary experiment. A control group (culture medium) and 6:2 FTSA exposure group (0.5 mg/L) were set up and were tested in triplicate for each group. Healthy zebrafish embryos (2 dpf) were randomly assigned to six Petri dishes, each containing 200 embryos and 30 mL of exposure solution. Exposed embryos continued to be incubated in a constant-temperature and -light incubator (28 ± 1 °C) to 5 dpf. Half of the exposure solution was renewed daily during the exposure period.

After exposure, zebrafish embryos were collected and repeatedly cleaned with PBS to remove residues. Then, 100, 50 and 40 embryos were randomly selected from each Petri dish, and immediately stored at −80 °C after being frozen in liquid nitrogen for later biochemical index detection, immune protein index detection and transcriptomic sequencing analysis, respectively.

### 2.4. Biochemical Index Detection

Zebrafish embryos (n = 300) were thoroughly ground and homogenized in PBS then centrifuged for 10 min (4 °C, 2500 rpm); the supernatant was removed for testing. Relevant indicators of oxidative stress (malondialdehyde (MDA), catalase (CAT) and superoxide dismutase (SOD)) and the inflammatory response (nitric oxide (NO), total and inducible NO synthase (TNOS and iNOS, respectively), lysozyme (LZM), acid phosphatase (ACP) and alkaline phosphatase (AKP)) were tested in strict accordance with the manufacturer’s instructions. Protein concentrations in each sample were determined using the Coomassie brilliant blue assay [29]. All biochemical indexes were measured in triplicate using a microplate reader.

### 2.5. Immune-Related Protein Expression

Zebrafish embryos (n = 150) were homogenized in precooled dilution buffer then centrifuged at 2500 rpm for 10 min at 4 °C; the supernatant was taken for measurements. The expression of immune-related proteins (interleukin-1β (IL-1β), tumor necrosis factor-α (TNF-α), nuclear factor-κB (NF-κB), Toll-like receptor 4 (TLR4)) in zebrafish embryos was detected using the enzyme-linked immunosorbent assay (Nanjing Jiancheng Institute of Biological Engineering, Nanjing, China) in strict accordance with the manufacturer’s instructions. All protein indexes were measured in triplicate using a microplate reader.

### 2.6. Transcriptomic Analysis

The RNA extraction, library construction and sequencing of zebrafish samples (n = 120) were completed by Hangzhou Lianchuan Biotechnology Co., Ltd. (Hangzhou, China). Briefly, total RNA was extracted using Trizol reagent (Thermo Fisher, Mississauga, ON, Canada). The total amount and purity of RNA were analyzed by a Bioanalyzer 2100 and an RNA 6000 Nano LabChip Kit (Agilent, Santa Clara, CA, USA), and high-quality RNA samples with an RNA integrity number > 7.0 were used for sequencing library construction. Finally, 2 × 150 bp paired-end sequencing (PE150) was performed on the Illumina Novaseq™ 6000 (Illumina, San Diego, CA, USA) in accordance with the manufacturer’s recommended protocol. To obtain high-quality data, low-quality reads were filtered out using Cutadapt software (https://cutadapt.readthedocs.io/en/stable/,version:cutadapt-1.9, accessed on 15–26 April 2022) and sequence quality was verified using FastQC software (http://www.bioinformatics.babraham.ac.uk/projects/fastqc/,0.11.9, accessed on 15–26 April 2022). Reads for all samples and comparisons to the zebrafish reference genome were completed using HISATA (version: hisat2-2.2.1) software. Gene abundance was quantified using StringTie, gffcompare and ballgown software, and fragments per kilobase per million transcripts (FPKM) values were calculated. Gene differential expression analysis was performed with DESeq2 software using false discovery rate parameters below 0.05 and an absolute fold change ≥ 2 to define differentially expressed genes (DEGs). The GO and KEGG databases were used to perform functional and pathway enrichment analyses of DEGs, respectively. Correlation and principal component analyses were performed using R software.

### 2.7. Statistical Analysis

All data are expressed as “mean ± standard deviation” (mean ± STD). Normality tests, one-way analysis of variance (ANOVA) and Tukey tests were performed to observe significant differences between the exposure and control groups using SPSS software. Origin software was used to draw and beautify the graphs. Differences were considered significant at * *p* < 0.05, ** *p* < 0.01, and *** *p* < 0.001. The number of independent parallel samples for each group in the experiment was ≥3.

## 3. Results

There were no deaths in either the control or experimental group during the exposure period, but morphological deformities were observed in the experimental group, mainly pericardial edema and yolk sac edema (Figures S1 and S2).

### 3.1. Effect of 6:2 FTSA on Oxidative Stress

**Figure 1:**
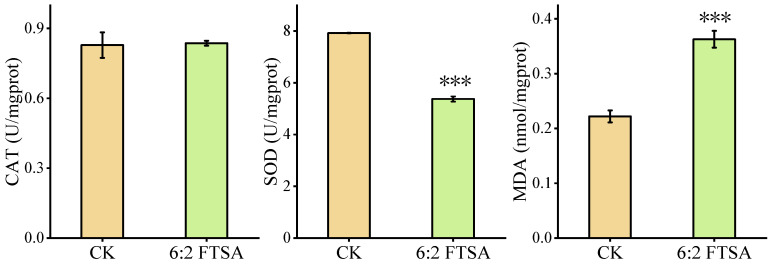
Effect of 6:2 FTSA exposure on CAT and SOD activities and MDA content in zebrafish. Data expressed as the mean ± standard deviation. Asterisks indicate significant differences between the exposure and control groups: *** *p* < 0.001. CK = control group; prot = protein; U = active unit.

Enzymatic activities of CAT and SOD and the concentration of MDA were measured in zebrafish embryos after exposure to 0.5 mg/L 6:2 FTSA for 72 h (Figure 1). Compared with the control group, the 6:2 FTSA exposure group showed a significant decrease in SOD activity and a significant increase in MDA content, but no difference in CAT activity. These results indicated that exposure to 0.5 mg/L 6:2 FTSA interfered with the antioxidant system of zebrafish embryos, inducing oxidative stress.

### 3.2. Effect of 6:2 FTSA on the Inflammatory Response

**Figure 2:**
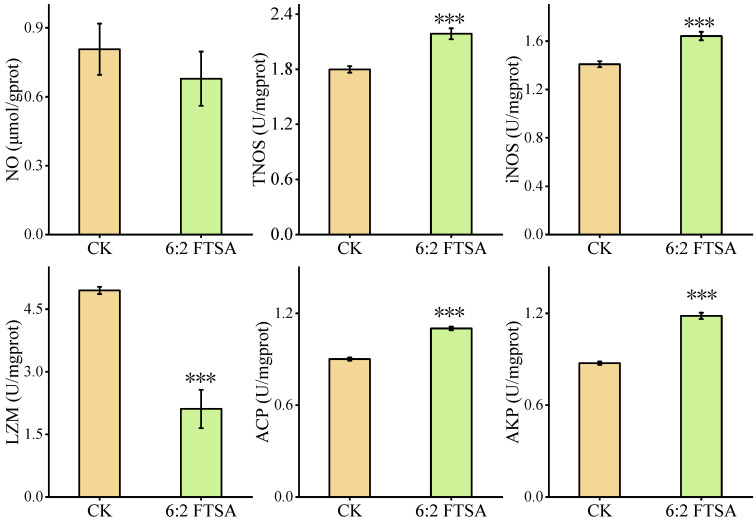
Effect of 6:2 FTSA exposure on TNOS, iNOS, LZM, AKP and ACP activities, and NO content in zebrafish embryos. Data expressed as the mean ± standard deviation. Asterisks indicate significant differences between the exposure and control groups: *** *p* < 0.001. CK = control group; prot = protein; U = active unit.

The inflammatory response-related factors NO, TNOS, iNOS, ACP, AKP and LZM were measured in zebrafish embryos after exposure to 0.5 mg/L 6:2 FTSA for 72 h (Figure 2). Compared with the control group, the 6:2 FTSA exposure group exhibited significant changes in all of these indicators except for NO. The activities of TNOS, iNOS, ACP and AKP were significantly increased, while that of LZM was significantly decreased, indicating that 6:2 FTSA exposure caused a disturbance in the inflammatory stress response in zebrafish embryos.

### 3.3. Effect of 6:2 FTSA on Immune-Related Proteins

**Figure 3:**
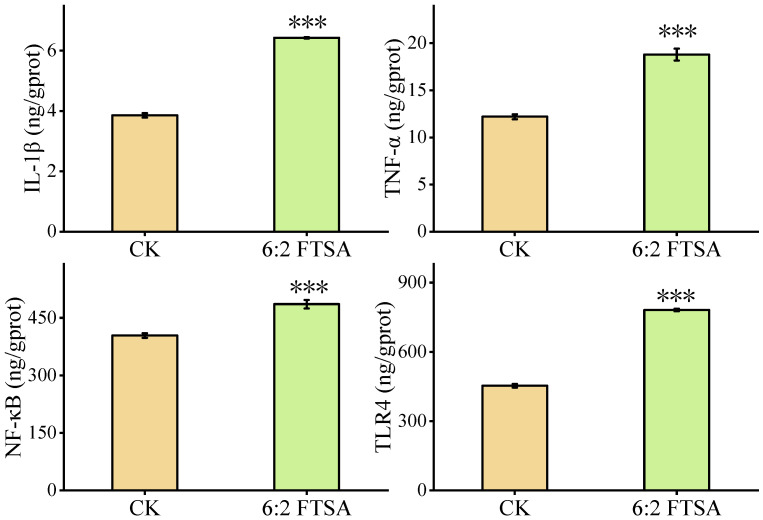
Effect of 6:2 FTSA exposure on IL-1β, TNF-α, NF-κB and TLR4 protein expression in zebrafish embryos. Data expressed as the mean ± standard deviation. Asterisks indicate significant differences between the exposure and control groups: *** *p* < 0.001. CK = control group; prot = protein.

The typical immune-related proteins IL-1β, TNF-α, NF-κB and TLR4 were also measured in zebrafish embryos after exposure to 0.5 mg/L 6:2 FTSA for 72 h (Figure 3). Compared with the control group, 6:2 FTSA exposure resulted in significantly higher expression levels of IL-1β, TNF-α, NF-κB and TLR4 in zebrafish embryos. These results suggested that 6:2 FTSA exposure amplified the inflammatory response in zebrafish embryos and induced organismal immunotoxicity.

### 3.4. Transcriptome Sequencing Analysis

**Figure 4:**
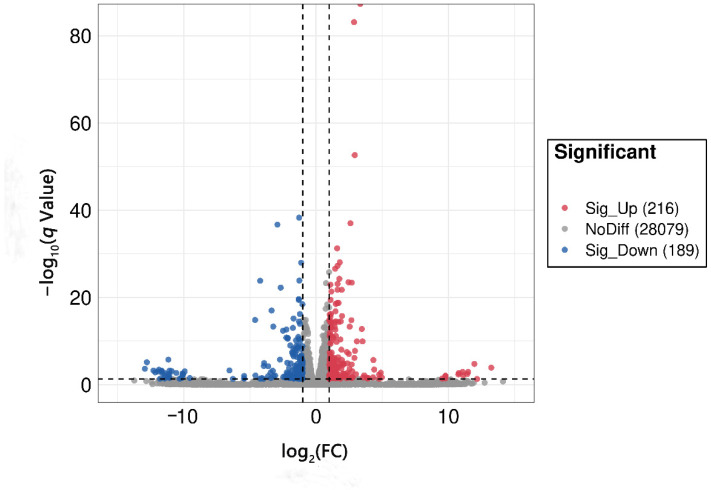
Volcano charts of DEGs after exposure to 6:2 FTSA in zebrafish embryos. FC = fold change.

**Figure 5:**
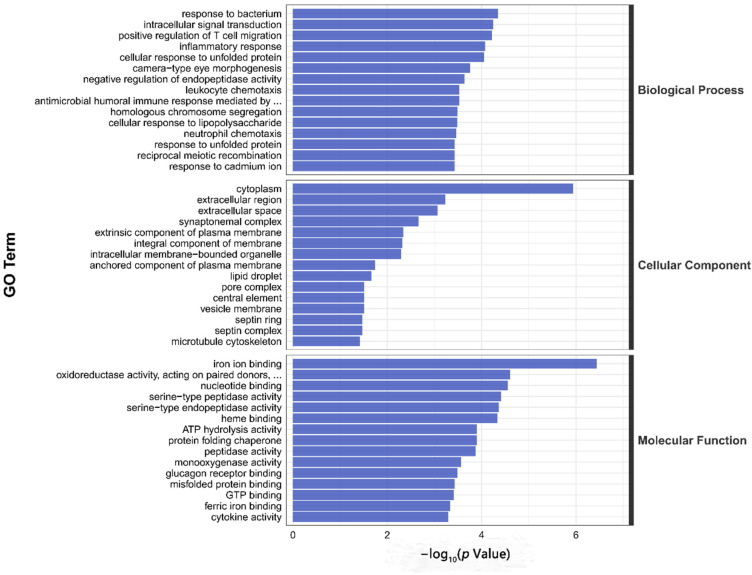
Histogram of GO enrichment classification of DEGs after exposure to 6:2 FTSA in zebrafish embryos.

**Figure 6:**
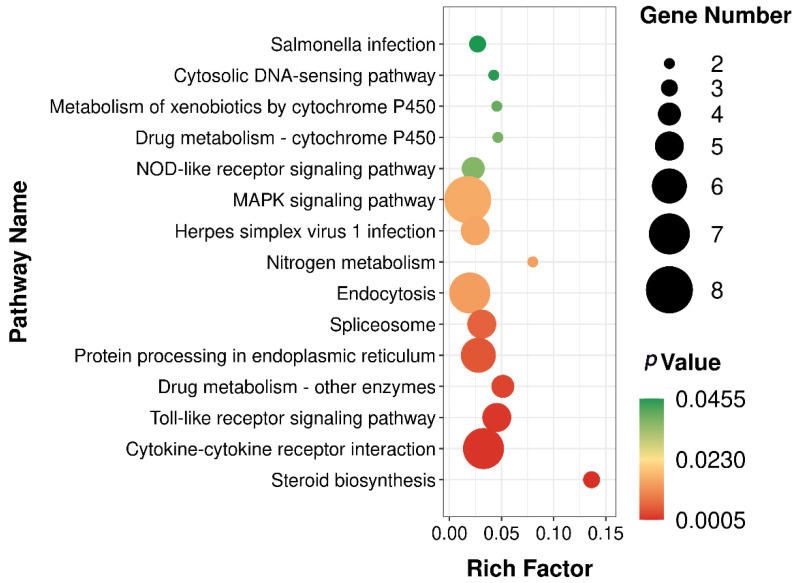
KEGG-enriched bubbles of DEGs after exposure to 6:2 FTSA in zebrafish embryos.

In our RNA sequencing data, the proportion of effective reads in each sample exceeded 90%, and the base quality scores, Q20 and Q30, indicated > 99.9% and 97.3% accuracy, respectively. The high-confidence data (clean data) matched the zebrafish species reference genome by up to 90%, and samples within each group yielded reproducible results, which satisfied the criteria for further analysis. DEG analysis showed that a total of 405 DEGs were screened in the 6:2 FTSA exposure group compared to the control group, of which 216 genes were upregulated and 189 genes were downregulated (Figure 4). Next, we analyzed the functions of these DEGs using the GO and KEGG databases. GO enrichment analysis found that the DEGs in 6:2 FTSA-exposed zebrafish embryos were mainly categorized as cell components (CC), biological processes (BP) and molecular functions (MF), with significant enrichment in 186 GO items, including the following: leukocyte migration and inflammatory response (GO: 0002523), nitric oxide synthase activity (GO: 0004517), inflammatory response (GO: 0006954) and leukocyte chemotaxis (GO: 0030595) (Figure 5). KEGG enrichment analysis found that the DEGs were significantly enriched in 15 pathways, including the mitogen-activated protein kinases (MAPK) signaling pathway (dre04010), cytokine receptor interaction (dre04060), Toll-like receptor (TLR) signaling pathway (dre04620) and NOD-like receptor (NLR) signaling pathway (dre04621) (Figure 6).

## 4. Discussion

Studies have shown that PFOS exposure can induce immunotoxicity in zebrafish [28,30,31]. Because PFOS and its substitute 6:2 FTSA share some structural similarity, we speculated that 6:2 FTSA may also interfere with the normal immune function of zebrafish and produce immunotoxicity. To investigate this hypothesis, we exposed zebrafish embryos at 2 dpf to a 0.5 mg/L 6:2 FTSA solution for 3 d and measured its effects on the innate immune response in this aquatic model organism.

Oxidative stress is the main mechanism of inducing body toxicity under exogenous compound stress. A large number of reactive oxygen species (ROS) cannot be effectively eliminated, thus causing lipid peroxidation [32]. As an end-product of lipid peroxidation, MDA is considered to indirectly reflect the degree of oxidative damage in the body [33]. In our study, 6:2 FTSA exposure caused a significant increase in MDA content in zebrafish embryos, indicating that 6:2 FTSA induced oxidative stress. Zou et al. found that exposure to PFOS and sodium *p*-perfluorous nonenoxybenzene sulfonate (OBS) also increased the contents of ROS and MDA in zebrafish embryos, causing oxidative damage [34]. Wu et al. also confirmed that exposure to the substitute 6:2 chlorinated polyfluorinated ether sulfonate (F-53B) induced toxicity similar to PFOS, not only for rapid accumulation in zebrafish larvae, but also for the induction of oxidative stress (reduced MDA content) [35]. This shows that PFOS and its substitutes have some similarity in inducing oxidative damage in zebrafish. The generation of oxidative damage means that the balance of oxidative and antioxidant systems in the body is broken. SOD and CAT, as the main antioxidant enzymes, play an important role in resisting oxidative stress [34]. While SOD uses free radicals as a substrate, converting O^2−^ to H_2_O_2_ and O^2^ and playing a dominant role in ROS clearance, CAT continuously converts H_2_O_2_ to H_2_O and O^2^, finally realizing the function of antioxidant damage [36]. Organisms exposed to pollutants usually respond by activating antioxidant enzyme activity. However, in our study, 6:2 FTSA exposure led a significant decrease in SOD activity and no significant change in CAT activity. The reason for this phenomenon may be that, at the early stage of the stress response, a large amount of SOD is consumed in the clearance of ROS, leading to a decrease in SOD activity [37,38]. CAT, as a scavenger of SOD product H_2_O_2_, requires two H_2_O_2_ molecules to meet CAT and collide on the active center in order to react [35]. Hence, the concentration of H_2_O_2_ produced by SOD transformation in the body directly limits the CAT decomposition rate. Therefore, the different synchronization between SOD and CAT may be due to the lag of induction of SOD to CAT. In addition, considering the similarity of antioxidant enzyme action, the H_2_O_2_ scavenging capacity of CAT may be replaced by glutathione peroxidase (GSH-Px), so the decrease in SOD activity did not result in significant changes in CAT activity [39]. For example, significant differences in SOD and GSH-Px activities were observed in zebrafish larvae after exposure to F-53B in the study by Wu et al. [40]. However, the GSH-Px index was not tested in this study, and the above speculations need to be further verified. Notably, significant increases in oxidative stress-related indicators (SOD, CAT, GSH-Px, ROS and MDA, etc.) in zebrafish larvae were found in both Du and Wang et al. with different concentrations (0.2–1.6 mg/L) of PFOS exposure (96 hpf) [41,42]. This represents a large difference from our 6:2 FTSA exposure results, and the reason for this difference may be related to the different enrichment capacity of the two. In our previous study, we found that although 6:2 FTSA could be enriched in zebrafish, its bioenrichment capacity was much weaker than that of PFOS, suggesting that PFOS tends to induce a faster process of toxic effects and exhibits a stronger biotoxicity under the same experimental conditions. Therefore, in order to prevent further aggravation of the degree of oxidative damage in the organism, SOD and CAT activities start to increase under PFOS exposure to enhance the tolerance of the organism to oxidative stress.

Studies have found that there is a complex correlation between oxidative stress and inflammatory response, mainly manifested as the occurrence of oxidative stress promoting the activation of the inflammatory response [43]. Because the occurrence of oxidative stress has been found in our previous studies on 6:2 FTSA exposure, we speculated that it would also interfere with the inflammatory regulatory process in zebrafish embryos. iNOS, a type of TNOS, is an early response enzyme in the inflammatory response. A large number of studies have shown that the inflammatory response is usually accompanied by the induction of iNOS [44]. As a free radical molecule, NO plays a central role in immune system damage and is mainly synthesized by iNOS [45]. In our study, although the activities of TNOS and iNOS were significantly increased under 6:2 FTSA exposure, the change in NO content was not significant. This may be due to the lag in the reaction between iNOS and NO. Similarly, no significant difference in NO levels under PFOS or OBS exposure was detected in the study by Huang et al. [43]. However, in a study by Liu et al., it was found that exposure to the PFOS substitute F-53B resulted in NO overload in zebrafish embryos/larvae, but iNOS activity did not change significantly and its gene expression levels were significantly upregulated, presumably because NO overload induced a negative feedback regulation in the organism [12]. Considering the variability of experimental conditions among different subject groups, the effects of different PFAS on the inflammatory response of zebrafish under the same conditions are yet to be established in order to facilitate the further screening of relatively safe alternatives to PFOS. Proteolytic enzymes ACP, AKP and LZM all belong to the main enzyme components of lysosomes and are considered to be reliable indicators for exogenous toxicological evaluation. LZM has an anti-inflammatory function and is the most important defense factor in fish innate immunity, directly reflecting the immunity level of zebrafish [28]. In our study, the increased activities of ACP and AKP indicated that they play an important role in the immune response, while the decrease in LZM activity may be attributable to its function as a main anti-inflammatory molecule in the response to the 6:2 FTSA stress, resulting in a large amount of consumption [46]. Similarly, in a study by Chen et al., it was found that 6:2 and 8:2 polyfluoroalkyl phosphate diesters not only accumulate and transform in carp, but also cause a significant increase in ACP and glutathione S-transferase (GST) activity in their liver tissues [47]. Wang et al. also observed a significant increase in AKP activity in serum and liver tissues of male Sprague Dawley rats under PFOA exposure and concluded that this substance could induce liver injury in the subject organisms [48]. Notably, Guo et al. investigated the effects of a concentration gradient (0, 0.02, 0.04, 0.08 mg/L) of PFOS exposure (7, 14, 21 d) on adult male zebrafish, and found that PFOS caused significant reductions in ACP, AKP and LSZ activity in the liver and severe impairment of the liver tissue microstructure [28]. In contrast to the direct immunosuppression by PFOS, our study found that 6:2 FTSA mainly induced the activation of immune protection in zebrafish embryos.

Immune proteins play important roles in fighting against exposure to xenobiotic compounds, and their content level in an organism can directly reflect physiological changes, thereby serving as key markers of the immunotoxicity of pollutants [49,50]. In the early development stage of the zebrafish embryo, only the innate immune system responds to external stimuli, with immune cells and immune cytokines providing the main regulatory functions in this process [12]. Such immune cytokines include chemokines, TNFs and ILs [43]. TLR4 is a transmembrane protein that plays a role in early pathogen recognition and innate immunity. Activation of this receptor induces the activation of downstream transcription factors such as NF-κB, which further enhances the production of pro-inflammatory factors including IL-1β and TNF-α, thereby promoting the inflammatory response to indirectly improve the immune response [51]. Huang et al. found that acute exposure to OBS (30 mg/L) significantly increased the expression levels of immune-related proteins (IL-1β, CXCL8, MMP9, Casp8) in zebrafish larvae, and was generally consistent with the transcriptomic results [43]. However, no abnormal expression of these proteins was observed in the PFOS (20 mg/L)-treated group, but a significant upregulation of the corresponding protein genes was detected, presumably due to the severe disruption of the translation process caused by PFOS exposure [43]. Similarly, it was also found that IL-1β protein levels in adult zebrafish liver tissue were reduced after long-term exposure (1 uM, 21 d) to PFOS, F-53B and OBS, with the most significant difference observed in the OBS-treated group [31]. Their findings highlighted that F-53B and OBS can also cause immunotoxicity similar to PFOS to some extent, and safety issues regarding the alternatives should be given due attention. Significant increases in immune-related protein indicators (NF-κB, TNF-α, IL-1β and TLR4) were detected in our study, indicating that 6:2 FTSA amplifies the inflammatory damage profile and induces organismal immunotoxicity in zebrafish larvae. This result further highlights that the health risk of 6:2 FTSA, another novel alternative to PFOS, should not be ignored. In previous studies, per- and poly-fluoroalkyl substance (PFAS)-induced inflammatory responses in fish were common, and the internal mechanism may be strongly correlated with TLR-NF-κB signaling pathway disorders. For example, the study by Guo et al. detected a significant increase in the concentration of NF-κB protein, suggesting that PFOS may activate the pro-inflammatory response of hepatocytes through the NF-κB signaling pathway, thus interfering with the liver immune regulation of adult zebrafish [28]. Yang et al. also found that enhanced NF-κB protein expression was consistent with the upregulation of most immune-related gene transcripts, suggesting that the NF-κB signaling pathway is involved in the process of F-53B-induced immunotoxicity in zebrafish larvae [44]. Additionally, Zhang et al. also pointed out that perfluorooctanoic acid (PFOA) exposure regulated the expression levels of cytokines (IL-1β, IL-4, IL-21 and interferon) in zebrafish immune organs (spleen and kidney) through the TLR/myd88/NF-κB pathway [52,53]. Likewise, our study detected significantly increased expression levels of key proteins (NF-κB, TNF-α, IL-1β and TLR4) in the TLR-NF-κB pathway, indicating that 6:2 FTSA induced an inflammatory response and may have improved the anti-inflammatory ability of zebrafish. This process may be regulated by the TLR-NF-κB pathway. Therefore, we speculated that 6:2 FTSA may have a similar immunotoxic mode of action to some PFAS.

To further explore the mechanism of immunotoxicity of 72 h of 6:2 FTSA exposure on zebrafish embryos, we conducted GO and KEGG enrichment analysis of DEGs. GO enrichment analysis showed that, compared with the control group, genes related to immune functions such as leukocyte chemotaxis, inflammatory response and humoral immune response were significantly enriched in the 6:2 FTSA exposure group, indicating that 6:2 FTSA may induce the inflammatory response and interfere with normal immune functioning in zebrafish. KEGG pathway analysis revealed significant changes in the TLR, NLR and MAPK signaling pathways, etc. These pathways are thought to play an important role in the regulation of immunotoxicity in zebrafish and are the focus of our toxic mechanisms in this study. Studies have indicated that the Nrf2-ARE signaling pathway can regulate the activity of antioxidant enzymes and play a protective role in the repair of oxidative damage in zebrafish [34]. Although the MDA and SOD indexes of zebrafish embryos in the 6:2 FTSA-exposed group were significantly changed in this study, no significant enrichment of this pathway was found in the KEGG enrichment analysis of DEGs. Further research is needed to determine how 6:2 FTSA exposure affects the signaling pathway and causes oxidative stress in zebrafish. TLRs are specific pattern recognition receptors that are used to identify various unique components of prokaryotes, fungi and pathogens, and the TLR signaling pathway plays an important role in the initiation of innate immunity [54,55]. NLRs, also part of the pattern recognition receptor subfamily, promote the inflammasome pathway (cytoplasmic signaling devices that activate caspase-1, IL-1β and IL-18), which can increase regulation of the immune response to pathogens and damaged self-molecules, as well as tissue repair [56,57,58]. The MAPK and NF-κB pathways both belong to the downstream signaling pathways of TLR and NLR, which can be stimulated and activated by TLRs and NLRs, thus increasing the expression of pro-inflammatory factors (e.g., IL-1β, TNF-α, CXCL8 and IL-12) and inducing inflammation [59,60]. The cytokines IL-1β and TNF-α, which are secreted by immune cells, also play key roles in activating neutrophils and other immune cells and promoting their recruitment to sites of inflammation [44]. Many studies have indicated that PFASs can affect the immune system of aquatic organisms, and the above signaling pathways may play a leading role in this process [28,53,61]. For example, the results of Tang et al. showed a positive correlation between the immunotoxic response induced by PFAS and carbon chain length, and that the TLR pathway plays an important role in the immunomodulation of PFAS in zebrafish [62]. Shi et al. found that acute exposure to PFOS affected the MAPK signaling pathway in zebrafish larvae, mainly by means of the significant upregulation of *JNK1*, *p38a* and *p38b* genes on this pathway, and that activation of nuclear factor erythroid 2–related factor 2 (Nrf2) could play an anti-toxicity role [63]. In our study, DEGs annotated to the TLR, NLR and MAPK signaling pathways included *hsp70*, *hsp701*, *stat1b*, *irf3*, *cxcl8b*, *map3k8*, *il1b*, *tnfa* and *nfkb*. Furthermore, the upregulated expression of some of these genes was consistent with the trend relating to the upregulated expression of the corresponding proteins, suggesting that upregulation of these genes activates the zebrafish antioxidant system and causes an inflammatory response. Therefore, we speculated that the TLR/NOD-MAPK signaling pathway played a major role in regulating the immune response of zebrafish embryos exposed to 6:2 FTSA. Notably, although we detected upregulation of *nfkb* expression and a significant increase in NF-κB protein expression, none of the DEGs were significantly enriched in the NF-κB signaling pathway, which is inconsistent with previous studies on the immune regulatory pathways involved in PFOS and PFOA exposure. The specific reasons for this remain to be explored. In addition, in this study, in addition to the immune-related pathways, we also noted significant differences in other virus-related pathways, which will also be the direction of our future research.

## 5. Conclusions

This study showed that 6:2 FTSA exposure affected antioxidant enzyme activity, caused lipid peroxidation, improved the activities of iNOS and various hydrolases, and promoted the inflammatory response in zebrafish embryos. GO and KEGG enrichment analyses of DEGs indicated that 6:2 FTSA exposure mainly caused changes in the expression of genes involved in the TLR/NOD-MAPK signaling pathway, leading to increased expression of the corresponding immune proteins, triggering the pro-inflammatory response, interfering with the normal immune function and causing immunotoxicity of zebrafish. This study broadens our understanding of the intrinsic mechanisms of 6:2 FTSA exposure in zebrafish embryos and provides theoretical data for the risk assessment of 6:2 FTSA in aquatic ecosystems.

## Data Availability

Data may be requested from the corresponding author if there is a reasonable demand.

## Supplementary Materials

The following supporting information can be downloaded at: https://www.mdpi.com/article/10.3390/toxics11050459/s1, Figure S1: Survival rate of zebrafish larvae under different treatment groups; Figure S2: Deformities of zebrafish larvae in different treatment groups.
