# Supplementary material for: Immunotoxicity and Transcriptome Analyses of Zebrafish (Danio rerio) Embryos Exposed to 6:2 FTSA
Source: Toxics. 2023 May 15;11(5):459. doi: 10.3390/toxics11050459 (PMC10222677; doi:10.3390/toxics11050459)
Supplement: Supplementary file 1 [file toxics-11-00459-s001.zip › toxics-2321428-supplementary.pdf]

Supplementary materials

Immunotoxicity and Transcriptome Analyses of Zebrafish (*Danio rerio*) Embryos Exposed to 6:2 FTSA

Jing Zhang, Zongming Ren * and Meng Chen *

Institute of Environment and Ecology, Shandong Normal University, Jinan 250358, China; 15506686558@163.com

* Correspondence: zmren@sdnu.edu.cn (Z.R.); chenmeng.1989@163.com (M.C.)

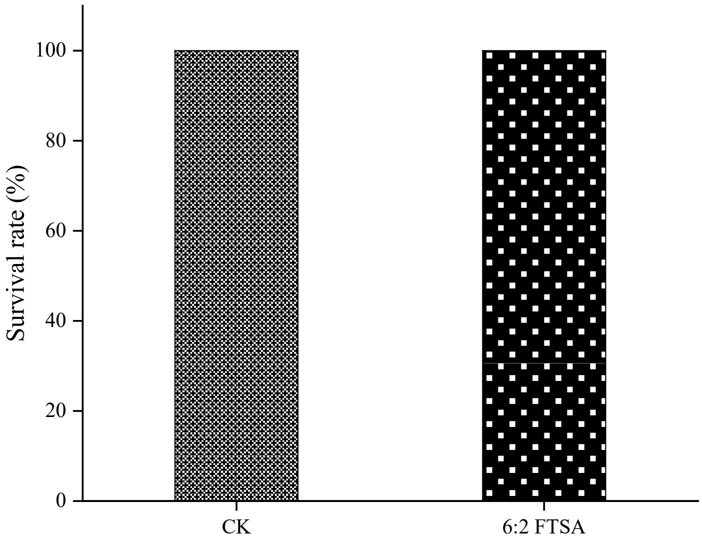

Figure S1. Survival rate of zebrafish larvae under different treatment groups. Data expressed as the mean ± standard deviation. CK=control group.

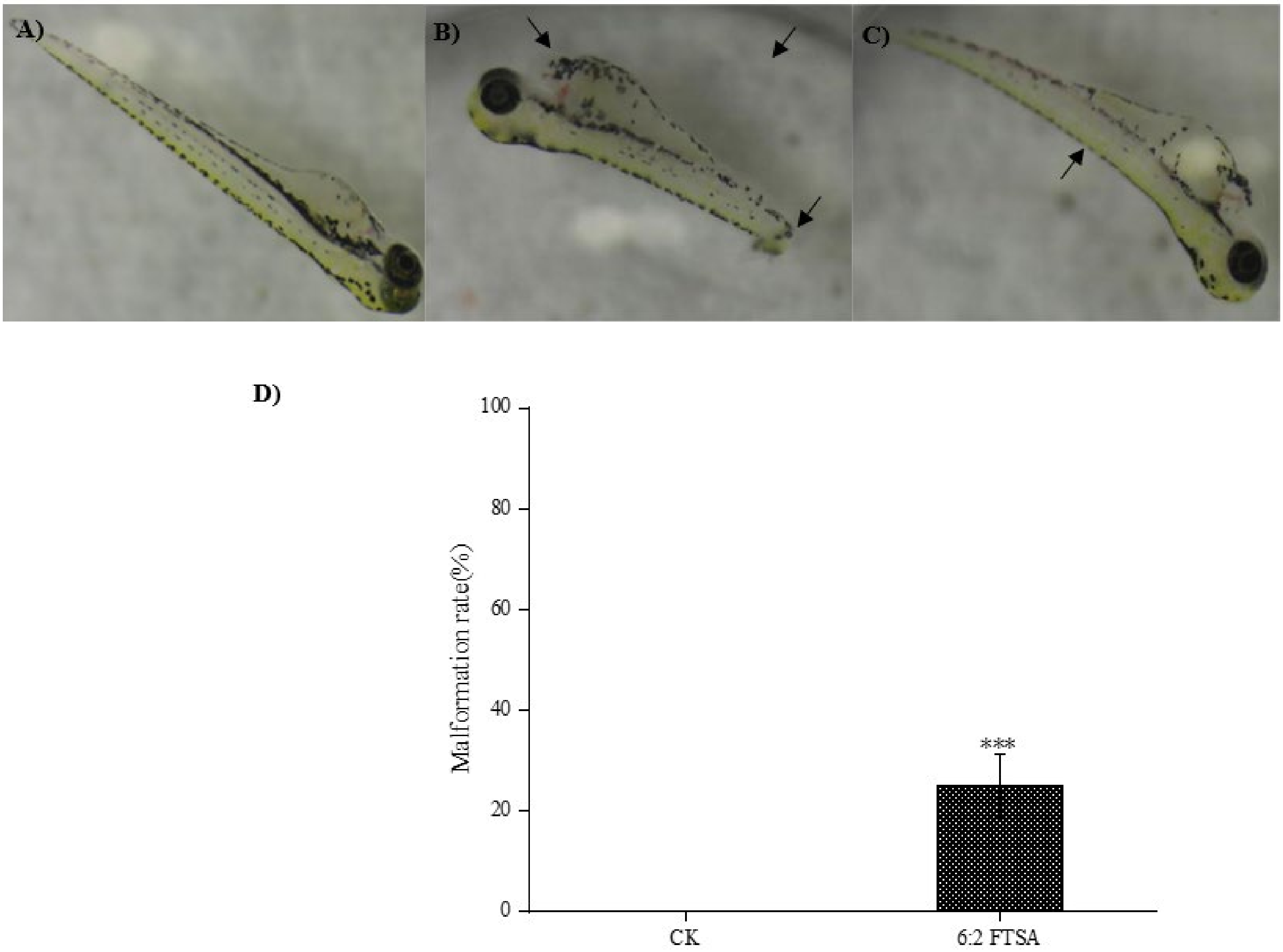

Figure S2. Deformities of zebrafish larvae in different treatment groups (**A**): normal; **B**): pericardial edema, yolk sac edema, tail curvature; **C**): scoliosis; **D**): Malformation rate). Data expressed as the mean ± standard deviation. Asterisks indicate significant differences between the exposure and control groups: ***$p < 0.001$. CK = control group.
